# A Pediatric Case of *Fusobacterium necrophorum* Mastoiditis and Meningitis Case Report in a Healthy Child and Review of the Literature

**DOI:** 10.1155/2024/6365796

**Published:** 2024-06-12

**Authors:** Elizabeth Feenstra, Aalt Van Roest, Juul Boes, Tom Spiritus, Sandra Kenis, Els L. I. M. Duval, Stephanie Vanden Bossche, Koen Vanden Driessche, Philippe G. Jorens

**Affiliations:** ^1^Pediatrics, (Pediatric) Critical Care, Pediatric Neurology, Radiology, General Internal Medicine, Infectious Diseases and Tropical Medicine, Antwerp University Hospital, Edegem, Belgium; ^2^Pediatrics and Microbiology, AZ Turnhout, Turnhout, Belgium; ^3^Laboratory of Experimental Medicine and Pediatrics (LEMP), University of Antwerp, Antwerp, Belgium

## Abstract

In infants and children, bacterial meningitis caused by anaerobic bacteria is rare. However, a serious infection with the anaerobe *Fusobacterium necrophorum* can occur in previously healthy children with a peak incidence in preschool children and in adolescents. As the clinical presentation can be very similar to meningitis caused by aerobic bacteria, one should consider *Fusobacterium necrophorum* as the causative agent when preceded by or associated with otitis media with purulent otorrhea or mastoiditis, in combination with minimal or no improvement on empiric antibiotic treatment. As this pathogen can be difficult to culture, anaerobic cultures should be obtained. Prompt treatment with a third-generation cephalosporin and metronidazole should be initiated once suspected or confirmed. Surgical source control is often necessary, but even with adequate and prompt treatment, the morbidity and mortality in children with a *Fusobacterium necrophorum* meningitis remains high. In this report, we describe a case of *Fusobacterium necrophorum* meningitis in a previously healthy child and review the available literature.

## 1. Introduction

In infants and children, a relatively small group of aerobic pathogens is responsible for most cases of bacterial meningitis. They vary by age, with *Streptococcus agalactiae*, *Escherichia coli*, and *Listeria monocytogenes* common in infants, while *Streptococcus pneumoniae*, *Neisseria meningitidis*, and *Haemophilus influenza* type B are most accountable for in children [1]. In contrast, bacterial meningitis caused by anaerobic bacteria in infants and children is rare, and the exact incidence is unknown. Different anaerobic bacteria have been reported, most frequently *Bacteroides* spp. (*fragilis*), *Clostridium* spp. (*perfringens*), and occasionally *Fusobacterium* spp. (*aquatile*, *gonidiaformans*, and *necrophorum*) [2–5]. In this report, we describe a case of a *Fusobacterium necrophorum* (*F. necrophorum*) meningitis in a previously healthy child and also review the available literature.

## 2. Case Report

A 3-year-old girl of Iranian descent, born in Belgium, with no significant medical history and immunized with the recommended vaccines, consulted a pediatrician because of left ear pain and fever for three days. Physical examination revealed a left acute otitis media, and amoxicillin was initiated. One week later, on day 10 of her illness, she presented at the emergency department because of persistent fever with anorexia, weight loss and night sweats. Physical examination revealed an erythematous nonbulging tympanic membrane on the left side without clinical signs of meningitis. Inflammatory parameters were slightly elevated with a C-reactive protein (CRP) of 40 mg/L (normal value: <3 mg/L). With these findings, an expectative management was set with a follow-up appointment in two days. Because of persistent fever, the girl was admitted for further diagnostic tests on day 12. Blood investigations now revealed a sedimentation rate of 57 mm/hour (normal value: <13 mm/hour), a white blood cell count of 14.7 × 10^9^/L (normal value: 4–12 × 10^9^/L), and a CRP of 22 mg/L. A nasopharyngeal aspirate was positive for adenovirus on a multiplex polymerase chain reaction (PCR) respiratory panel. Chest X-ray was unremarkable and blood- and urine cultures remained negative. Despite persistent fever, she was generally well and on the second day of admission her parents choose for an ambulatory follow-up.

Two days later she represented again in the emergency department because of high-grade fever, vomiting, and drowsiness. On admission, the girl was lethargic with symmetric but nonresponsive pupils, intermittent nystagmus, and eye deviation to the right. The left tympanic membrane was erythematous and bulging. A computed tomography scan of the brain revealed a mild hydrocephalus with an acute otitis media and mastoiditis on the left side. Blood investigation showed a normal white blood cell count of 7.3 × 10^9^/L and highly elevated CRP of 319 mg/L. The cerebrospinal fluid (CSF) obtained by lumbar puncture was purulent and revealed a leukocytosis of 345.000/*μ*L (normal value: <5 leukocytes/*μ*L), a low glucose value of less than 5 mg/dL (normal value: 40–80 mg/dL), and a high protein concentration of 1836 mg/dL (normal value: 5–40 mg/dL). Urgent treatment for bacterial meningitis was initiated with intravenous cefotaxime and dexamethasone. She was transferred to our university hospital for admission on the pediatric intensive care unit (PICU).

**Figure 1:**
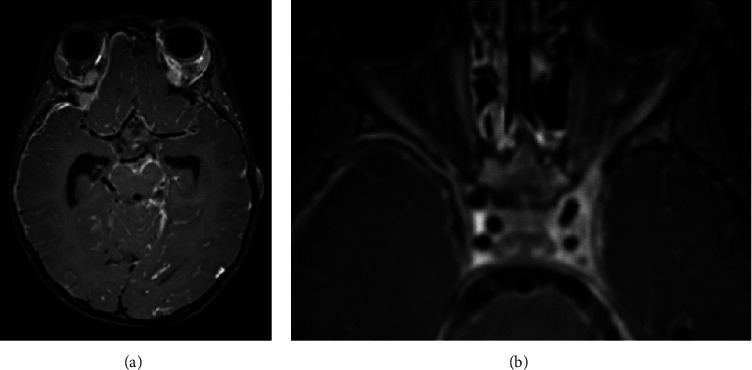
MRI day 1. Vessel wall imaging (axial 3D T1 TSE SPACE Dante) after IV gadolinium administration. (a) There is diffuse supratentorial and infratentorial pathological leptomeningeal contrast enhancement compatible with meningitis. Also note the pathological contrast enhancement of the walls of the cerebral arteries, indicative of associated vasculitis. (b) The cavernous sinus appears thickened on the left side and enhances more heterogeneously, suggestive of inflammation. There is secondary narrowing of the left cavernous carotid artery.

**Figure 2:**
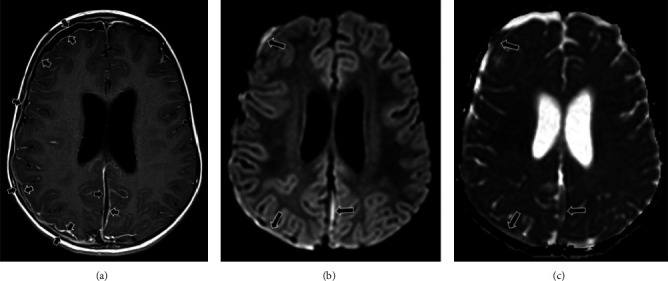
MRI day 1. Axial postcontrast T1 TSE SPACE (a), b1000 diffusion-weighted image (b), and ADC map (c). There is a right-sided and an interhemispheric subdural empyema, characterized by pathological pachymeningeal and leptomeningeal contrast enhancement (short arrows), and increased diffusion restriction (long arrows). Also, note the mild hydrocephalus.

**Figure 3:**
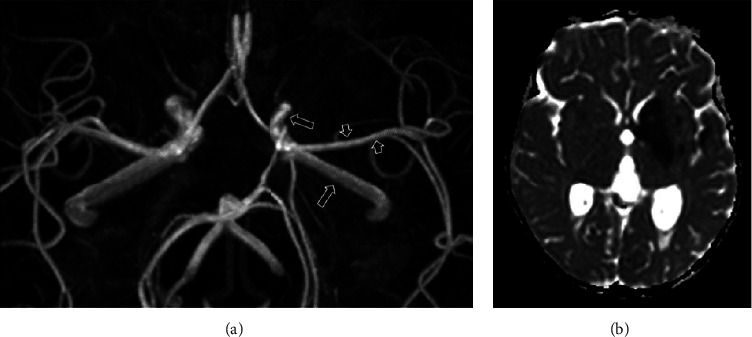
MRI day 1. Axial maximum intensity projection (MIP) of the 3D time of flight MR angiography (3D TOF MRA) (a) and ADC map at the level of the basal ganglia (b). The left internal carotid artery (long arrows) and proximal middle cerebral artery (short arrows) are narrowed on the 3D TOF MRA MIP reconstruction. There is a secondary ischemic infarct in the left basal ganglia, insular region, and temporal lobe, seen as low signal on the ADC map.

**Figure 4:**
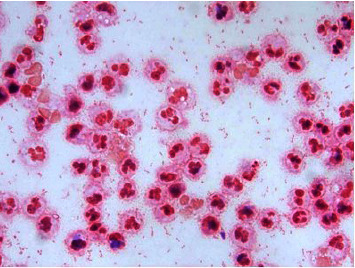
Gram stain of the cerebrospinal fluid with multiple Gram-negative bacilli clearly visible.

**Figure 5:**
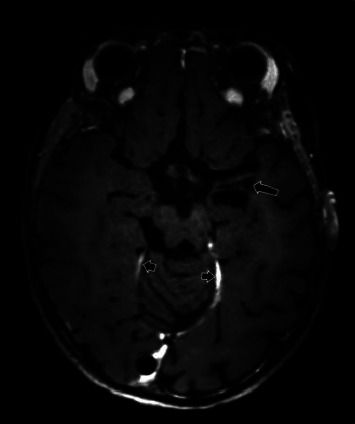
Follow-up MRI 18 months later. Vessel wall imaging (axial 3D T1 TSE SPACE Dante) after IV gadolinium administration. The diffuse leptomeningeal contrast enhancement and vessel wall contrast enhancement are no longer visible. There is limited residual contrast enhancement of the cerebellar tentorium (short arrow), and focal cystic encefalomalacia in the left mesial temporal lobe (long arrow) and basal ganglia (not shown) after ischemic infarction.

Shortly after admission at the PICU, further neurological deterioration was observed with increased drowsiness and decerebrate posturing of the right arm on pain stimuli. Furthermore, persistent eye deviation and hypertonia of the right arm and leg was noted and with the suspicion of convulsions treatment with levetiracetam was started. She was intubated and an urgent MRI of the brain revealed diffuse meningitis accompanied by a subdural empyema over the right cerebral convexity and against the cerebral falx with additional signs of ventriculitis with hydrocephalus and periventricular edema. The MRI also showed vascular involvement of the left cavernous sinus and the circle of Willis, which resulted in ischemic infarcts most notably in the left middle cerebral artery territory (Figures 1–3). Besides signs of left mastoiditis, contrast enhancement of the left os petrosum was noted. The Gram stain of the liquor revealed Gram-negative bacilli (Figure 4) and multiplex PCR meningitis/encephalitis panel that targets 14 of the most common bacterial, viral, and fungal causes were negative. To cover for anaerobic and resistant Gram-negative organisms, therapy was switched to meropenem and initially also amikacin. CSF culture became positive one day later and *F. necrophorum* was identified with matrix-assisted laser desorption/ionization time of flight (MALDI-TOF). The antibiotic regimen was switched to ceftriaxone and metronidazole. Blood cultures remained negative. A lumbar puncture was repeated on day three and revealed a raised intracranial pressure of 25 cmH_2_O (normal value: <20 cm H_2_O) and still purulent CSF. An external ventricular drain in the right ventricle was placed and a left tympanocentesis with placement of a tympanostomy tube for source control was performed. The CSF and purulent middle ear fluid cultures obtained during these procedures remained negative. Between day two and day four, after switching to antibiotics with strong effectivity against anaerobes, the patient remained afebrile and CRP decreased from 411 mg/L to 88 mg/L. However, on day five, the fever recurred. CT of the brain showed increasing opacification of the left middle ear and mastoid, and a left mastoidectomy was performed. Despite only low doses of continuous midazolam in the first 48 hours of admission, she remained comatose with roving eye movements, mid-dilated unresponsive pupils, absent tendon reflexes, and asymmetric hypertonicity on the right side. Although patient-triggered breaths were noticed, she could not be weaned from the ventilator in the first week after admission. On day 12, a new brain MRI showed a reduction of the diffuse leptomeningeal enhancement, vessel wall enhancement, and cavernous sinus inflammation. However, there was a slight increase of the subdural empyema, increase of ventriculitis, and a new ischemic infarction in the right globus pallidus. With these findings, a collaborative decision was made with the team to restrict further surgical therapy (craniotomy for neurosurgical drainage of the subdural empyema) since a poor prognosis was to be expected. However, the neurological situation slightly improved in the end of the second week and extubation was successful on day 14. From day five till 16, the patient remained afebrile and CRP gradually decreased to 15 mg/L. The following weeks, the patient had intermittent low-grade fever with fluctuating CRP between 15 and 48 mg/L. MRI repeated on day 23 showed a slight decrease of the subdural empyema. Treatment with ceftriaxone and metronidazole was continued. Another MRI after 6.5 weeks treatment with intravenous ceftriaxone and metronidazole, showed a minimal right-sided residual empyema in the posterior fossa (Figure 5) and signs of petrous apicitis (mastoiditis of the apex of the os petrosum). Treatment was extended with another three weeks of oral doxycycline. The patient was discharged from the hospital to a rehabilitation center on day 59.

Clinical neurological evaluation seven months after onset of the infection revealed a right central facial paresis and a severe central motor disorder type with right sided spastic-dystonia hemiplegia and moderate psychomotor delay.

## 3. Discussion

*F. necrophorum* is an anaerobic Gram-negative rod-shaped bacterium: in total 35 species of *Fusobacterium* have been identified. *F. necrophorum* is commonly present in the microbiome of the upper respiratory tract. Nevertheless, it may cause local infections such as pharyngitis, tonsillitis, and otitis which can progress to more invasive infections such as mastoiditis, osteomyelitis, or septicemia. A complication of a *F. necrophorum* infection is Lemierre's syndrome in which a local infection in the oropharyngeal cavity is leading to septic thrombophlebitis with thrombosis in the internal jugular vein, followed by spread of septic emboli mostly to the lungs, liver, or joints [6]. Meningitis is a rare complication.  Table 1 provides an overview of all 39 previously published cases of *F. necrophorum* meningitis in children until the age of 18 years [7–32].

The pathogenic mechanism leading from *F. necrophorum* asymptomatic upper respiratory tract colonization to infection is not well defined. Studies have revealed a multifactorial pathogenesis including patient-specific contributions by host, organism, and environmental factors [33]. In Lemierre's syndrome, the primary infection spreads from the oropharyngeal mucosa into the lateral pharyngeal space and soft tissues of the neck, and two different pathogenic routes are proposed. Either direct invasion through the connective tissue and lymphatic or hematogenous spread [34]. After the infection reaches the pharyngeal space and soft tissues, *F. necrophorum* can cause thrombosis in the smaller veins with extension to the internal jugular vein and sometimes retrograde extension to the sigmoid sinus. In meningitis, prolonged inflammation and edema can lead to septic thrombophlebitis even with the release of emboli into the systemic circulation [35]. Beside the classical Lemierre's syndrome, the otogenic variant of Lemierre's syndrome is also reported in children [23, 36]. In otogenic Lemierre's syndrome, a local infection spreads the mastoid through direct invasion or hematogenous spread to the cerebral venous sinus causing (septic) thrombophlebitis.

Most cases of *F. necrophorum* meningitis occurred in previously healthy children. Jacobs et al. reported a history of a previous car accident without lesions [13] and Westhout et al. an aseptic meningitis [22], respectively, two months and two years before the meningitis making a causative relation less likely. In many patients, including ours, there is often no visible destruction of the bony wall of the middle ear, suggesting hematogenous spread of the infection to the meninges. Destruction of the bony wall of the middle ear in the cases with a mastoiditis is not reported. However, in our case, on the first MRI of the brain contrast enhancement of the os petrosum was noticed, suggesting extensive inflammation from the mastoid evolving to suppurative inflammation of the petrous apex [37], which was noted on the third MRI. This petrous apicitis can occur if an infection of the middle ear and mastoid extends into the previously pneumatized petrous apex air cells. The location of the petrous apex is the most medial of the os temporale and near important intracranial structures under which the cavernous sinus and the meninges. In our patient, there were signs of inflammation of the cavernous sinus which suggests direct invasion from the mastoid and the os petrosum into the cavernous sinus and the meninges.

Children with *F. necrophorum* meningitis will mostly present with nonspecific signs such as high-grade fever, headache, and neck stiffness. These symptoms are the same as in any form of meningitis (bacterial or viral) and therefore no differentiation can be made based on clinical presentation only. However, the likelihood of a *F. necrophorum* meningitis increases if the clinical presentation is preceded by a recent upper respiratory tract infection, especially otitis media with purulent otorrhea, generally with minimal or no improvement on first line antibiotics. This was also applicable in our case and recognition of this clinical feature in patients presenting with meningitis can be an argument for the early association of antibiotics covering anaerobic pathogens.

**Figure 6:**
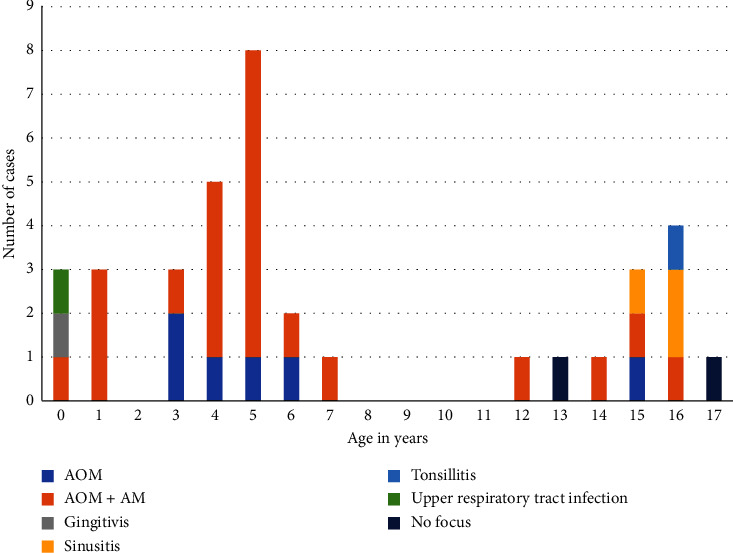
Distribution of age and primary infection site in children with *F. necrophorum* meningitis.

Review of the literature revealed an age distribution with two peaks in incidence in children with *F. necrophorum* meningitis (Figure 6): the first peak occurred in preschool children (mean age of 3 years and 10 months old, range 0.2–7 years) and a second peak in adolescents (mean age of 15 years old, range 12–17 years). The primary infection in the younger children was otitis media or mastoiditis. The primary infection in the adolescents seemed more diverse as sinusitis and a peritonsillar abscess were noted as primary infection too. Our data are supported by Yarden-Bilavsky et al. who respectively noted a median age of 2 years and 2 months and 1 year and 7 months in children with a mastoiditis caused by *F. necrophorum* [38, 39]. Our patient being three years old had an acute otitis media with mastoiditis.

*F. necrophorum* meningitis can be a challenging clinical diagnosis, complicated by the difficulties in isolating and identification of the bacteria itself. Current pediatric infectious disease guidelines recommend only routinely aerobic blood cultures, and anaerobic blood cultures should only be obtained when clinically relevant [40, 41]. Moreover, smaller pediatric blood culture bottles are often used which support the growth of most common pathogens with a small blood volume but only intended for aerobic growth. *F. necrophorum* only grows under strict anaerobic conditions. Furthermore, if anaerobic cultures are inoculated, the growth of *F. necrophorum* is often slow and sometimes the organisms fail to grow. Marcellus et al. reported a positive blood culture for *F. necrophorum* and the CSF stain showed Gram-negative bacilli, however the CSF culture remained negative [31]. If growth succeeds, it often takes days before identification of *F. necrophorum* is possible; Jensen reported a median time of three to four days [42], while Hagelskjaer Kristensen and Prag reported a median time up to six days [43]. In our patient, the diagnosis was suspected when the CSF revealed Gram-negative rods, with a definitive identification of *F. necrophorum* one day later. Despite the clinical severity, blood cultures obtained before the start the antibiotics treatment remained negative. In 17 out of the 29 cases who reported the results of the specific cultures, the diagnosis of a *F. necrophorum* meningitis was also confirmed by a positive CSF culture. In the 12 other cases, the diagnosis was confirmed by a positive blood culture in combination with clinical signs of meningitis and a positive CSF analysis with pleocytosis, high protein, and low glucose level.

Infants (outside the neonatal period) and children in suspicion of meningitis are often empirically treated with a third-generation cephalosporin (cefotaxime or ceftriaxone) with or without vancomycin [44, 45]. These antibiotics cover the most prevalent aerobic bacteria of meningitis in this age category, such as *S. pneumoniae*, *H. influenzae*, and *N. meningitidis*. In our patient, cefotaxime was started and when the Gram stain of the CSF showed Gram-negative bacilli, therapy was switched to meropenem in suspicion of an anaerobic cause. One day later, *F. necrophorum* was identified and treatment was amended to metronidazole and ceftriaxone. In the past decades, the antimicrobial resistance among anaerobes including *F. necrophorum* has increased making the susceptibility less predictable. Due to the production of beta-lactamase by some isolates, treatment with penicillin, ampicillin, amoxicillin and piperacillin are limited and beta-lactamase inhibitors such as clavulanate, sulbactam, or tazobactam should be added to the therapy [46]. Other susceptible antibiotics for *F. necrophorum* are carbapenem, clindamycin, or metronidazole. However, in case of *F. necrophorum* meningitis, treatment with a beta-lactam beta-lactamase inhibitor is not sufficient because of the poor blood-brain barrier penetration of the beta-lactamase inhibitors. Metronidazole is a specific anaerobic antibiotic with the advantages of a good blood-brain barrier penetration and oral availability and therefore a susceptible treatment in case of *F. necrophorum* meningitis [47]. Since metronidazole only demonstrates anaerobic activity, the association of an antibiotic with aerobic effect remains necessary since polymicrobial infection might occur in anaerobic infections [46]. In our review 23 authors reported the specific antibiotic treatment. In four cases metronidazole was started as initial therapy, two of these patients had a full recovery and two recovered with morbidities. In 13 cases, metronidazole was added to the initial therapy, five of these patients had full recovery, four recovered with morbidities, and three died. In six patients metronidazole was not used. As initial therapy in the latter, four of these patients started with cephalosporin/±amikacin, one patient with penicillin/amikacin, and one with meropenem. Of these patients, five died due to complications caused by the *F. necrophorum* meningitis. Almost all reported patients with *F. necrophorum* meningitis, including ours, underwent source control surgery, such as the placement of tympanostomy tubes, mastoidectomy, or endoscopic/surgical drainage of extra- or intracranial abscesses or empyema. Despite adequate treatment and early source control, the mortality and morbidity in children with a *F. necrophorum* meningitis remains high. Of the 27 patients, for which the authors reported the outcome, eight patients died and 11 patients, including our patient, recovered with morbidities. Chronical sequelae vary from mild neurological complications such as a moderate Horner syndrome to severe neurological impairment such as spastic hemiplegia.

## 4. Conclusion

Meningitis caused by *F. necrophorum* is a rare but serious infection which can occur in previously healthy children mostly preschool children and adolescents. As the clinical presentation and the laboratory findings can be very similar to another meningitis caused by aerobic bacteria, a meningitis preceded by or associated with otitis media or mastoiditis should raise suspicion for *F. necrophorum* meningitis. Specifically, when there is minimal or no improvement on empiric (often antiaerobic) antibiotic treatment. When a *F. necrophorum* meningitis is being suspected, it is important to obtain anaerobic cultures and to keep in mind that this pathogen can be difficult to culture. A negative culture does not rule out *F. necrophorum* meningitis. Prompt treatment with a third-generation cephalosporin and metronidazole are being recommended and surgical source control is often necessary. However, even with adequate treatment, the morbidity and mortality in children with *F. necrophorum* meningitis remains high.

## Figures and Tables

**Table 1:** Overview of all reported cases of *F. necrophorum* meningitis under the age of 18 years.

	Author	Year	Age/sex	Primary infection	Positive culture(s)	Treatment	Specific ATBT		Total duration ATBT	Outcome
1	Islam	1980	13/F	No focus	CSF (postmortem)	ATBT	AMP + PEN		N/A	Death

2	Adams	1983	16/F	Sphenoidal sinusitis	Blood	ATBT, surgical drainage of sphenoidal sinuses	PEN MTZ + PEN	3 d 42 d	6 weeks	Moderate left-sided Horner syndrome

3	Reynolds	1985	6/M	AOM	CSF, blood, middle ear fluid	ATBT, MD	CHL + PEN CHL + MTZ + PEN		N/A	Right-sided spastic hemiplegia, right-sided homonymous hemianopia, educational difficulties

4	Bouvier	1985	17/M	No focus	Blood	ATBT	AMP + MTZ MTZ	N/A 28 d	4 weeks	Full recovery

5	Tarnvik	1986	4/F	AOM	CSF, middle ear fluid	ATBT, TS tubes	CXM ERY ERY + MTZ	1 d 2 d 8 d	±2 weeks	Full recovery

6	Pace-Balzan	1991	5/F	AOM + AM	Blood	ATBT, MD	CHL + PEN CHL + MTZ + PEN MTZ + PEN	2 d 8 d 21 d	±4 weeks	Palsy of lateral *m. rectus*

7	Jacobs	1993	5/F	AOM	CSF	ATBT	CTX CHL + CRO AMC + TOB	2 d 1 d 2 d	N/A	Death

8	Bader-Meunier	1994	5/M	AOM + AM	CSF, blood, middle ear fluid	ATBT, MD	CTX CTX + MTZ MTZ oral	4 d 21 d 60 d	12 weeks	Full recovery

9	Figueras	1995	5/F	AOM + AM	CSF, blood	ATBT, TS tubes	CTX CTX + PEN CTX + MTZ + PEN	3 d 15 d 21 d	5.5 weeks	Full recovery

10	Larssen	1997	9 m/M	URTI	CSF	ATBT, ventriculostomy	AMP + CTX		N/A	Death

11	Voie	2002	14/M	AOM + AM	Blood	ATBT, MD	CHL + PEN MTZ + PEN MTZ oral	3 d 12 d 28 d	6 weeks	Hypoglossus paresis

12	Morrison	2004	15/F	AOM + AM	CSF, blood, middle ear fluid	ATBT, MD	CAZ + MTZ + VAN MTZ + PEN	14 d 28 d	6 weeks	Sensorineural hearing loss

13	Bentham	2004	15/M	Sphenoidal sinusitis	Blood	ATBT, surgical drainage of sphenoidal and ethmoidal sinuses	CRO + MTZ + VAN MTZ		6 weeks	Spastic left hemiparesis, left homonymous hemianopia

14	Veldhoen	2007	4/M	AOM + AM	CSF, blood	ATBT, TS tubes, surgical drainage of ventricular empyema	CRO MEM	1 d	N/A	Death

15	Veldhoen	2007	5/M	AOM + AM	CSF, blood	ATBT, MD	MEM		N/A	Death

16	Duquesne	2007	11 m/N/A	Gingivitis	CSF	ATBT	AMK + CTX + VAN AMX + MTZ	2 d	N/A	Death

17	Westhout	2007	16/M	Peritonsillar abscess	Blood	ATBT, surgical drainage of peritonsillar abscess with tonsillectomy, myringotomy	AZM + CLI + CRO CRO + MTZ CRO + MTZ + VAN CRO + MTZ + PEN CRO + PEN		12 weeks	Full recovery

18–21	Le Monnier	2008	1.3 (0.2–1.8) 2 M/2F	AOM + AM	CSF (2/4), blood (4/4), middle ear fluid (1/4), retro auricular abscess (4/4)	ATBT, MD (2/4)	N/A		9.7 weeks (4 weeks–16.5 weeks)	N/A

22	Vincent	2010	5/F	AOM + AM	Blood	ATBT	AMX + MTZ AMX oral	28 d	N/A	Full recovery

23	Angelino	2012	15/M	AOM	Blood	ATBT	AMK + CRO MTZ	2 d	N/A	Death

24	Megged	2013	3/F	AOM	CSF	N/A	N/A		N/A	Full recovery

25	Van Munster	2013	3/F	AOM + AM	CSF	ATBT, MD, surgical drainage of cerebellar empyema	CRO PEN			Eye and balance problems

26	DeGaffe	2013	12/M	AOM + AM	Blood	ATBT, TS tubes, MD	CRO + VAN FEP + MTZ + VAN	1 d 42 d	6 weeks	Full recovery

27–29	Shamriz	2015	N/A	N/A	N/A	N/A	N/A		N/A	N/A

30	Burgess	2017	3/M	AOM	Intracranial	ATBT, surgical drainage of epidural empyema	N/A		N/A	Ankyloses of temporomandibular joint

31	De Marcellus	2021	16/M	Sphenoidal sinusitis	Blood	ATBT, endoscopic drainage	CTX CTX + LVX + MTZ		N/A	Death

32–39	Thevis	2022	16/M 7/M 4/M 5/F 4/M 6/M 4/F 5/M	AOM + AM AOM + AM AOM + AM AOM + AM AOM + AM AOM + AM AOM + AM AOM + AM	N/A	ATBT, decompress craniotomy ATBT, TS tubes ATBT, TS tubes, MD ATBT, TS tubes ATBT, TS tubes, MD ATBT ATBT, TS tubes, MD ATBT, TS tubes, MD	CAZ + CLI + MTZ + PEN CRO CLI + CRO CRO + MTZ CLI + CRO + MTZ CAZ + MTZ + PEN CLI + CRO + MTZ CLI + CRO + MTZ + PEN		N/A N/A N/A N/A N/A N/A N/A N/A	Symptomatic epileptic insults N/A N/A Functional impairment left arm N/A N/A N/A N/A

40	Feenstra	2024	5/F	AOM + AM	CSF	ATBT, MD, external ventricular drain	CTX AMK + MEM CRO + MTZ DOX oral	1 d 1 d 50 d 21 d	10.5 weeks	Severe central motor disorder with right sided spastic-dystonia hemiplegia, right central facial paresis, moderate psychomotor delay

AM = acute mastoiditis; AMC = amoxicillin-clavulanate; AMK = amikacin; AMP = ampicillin; AMX = amoxicillin; AOM = acute otitis media; ATBT = antibiotic treatment; AZM = azithromycin; CAZ = ceftazidime; CHL = chloramphenicol; CLI = clindamycin; CRO = ceftriaxone; CSF = cerebrospinal fluid; CTX = cefotaxime; CXM = cefuroxime; DOX = doxycycline; ERY = erythromycin; FEP = cefepime; LVX = levofloxacin; MD = mastoidectomy; MEM = meropenem; MTZ = metronidazole; N/A = not available; PEN = penicillin; TS = tympanostomy; VAN = vancomycin.

## Data Availability

The datasets analyzed during the current study are available from the corresponding author on reasonable request.
